# Supplementary material for: Coherent tunneling in an AlGaN/AlN/GaN heterojunction captured through an analogy with a MOS contact
Source: Sci Rep. 2017 Aug 15;7:8177. doi: 10.1038/s41598-017-08307-0 (PMC5557989; doi:10.1038/s41598-017-08307-0)
Supplement: Supplementary file 1 — Supplementary information [file 41598_2017_8307_MOESM1_ESM.pdf]

Supplementary Information

"Coherent tunneling in an AlGaN/AlN/GaN heterojunction captured through an analogy with a MOS contact"

Y. Baines, J. Buckley, J. Biscarrat, G. Garnier, M. Charles, W. Vandendaele, C. Gillot and M. Plissonnier.

February 22, 2017.

Review: Scientific Reports [Nature Publishing Group]

The following supplementary information document details different material parameters and equations entering the calculation of the conduction band profile at the vicinity of the diode's anode contact and the transport properties.

Additionally a comparison between the calculation of the forward conduction current using the WKB approximation and the Transfer Matrix formalism is given.

III-N band gap parameters

GaN electron affinity: $\chi^{GaN} = 4.1\ eV$

GaN energy band gap: $E_g^{GaN} = 3.55\ eV$

AlN energy band gap: $E_g^{AlN} = 6.06\ eV$

$Al_xGa_{1-x}N$ energy band gap [1]: $E_g^{Al_xGa_{1-x}N}(x) = xE_g^{AlN} + (1-x)E_g^{GaN} - x(1-x)$

$Al_xGa_{1-x}N$ vs GaN conduction band offset [1]: $\Delta E_c(x) = 0.7(E_g^{Al_xGa_{1-x}N}(x) - E_g^{GaN})$

III-N Spontaneous polarization

GaN spontaneous polarization charge [1]: $P_{sp}^{GaN} = 0.029\ C/m^2$

AlN spontaneous polarization charge [1]: $P_{sp}^{AlN} = 0.081\ C/m^2$

$Al_xGa_{1-x}N$ spontaneous polarization charge: $P_{sp}^{Al_xGa_{1-x}N} = xP_{sp}^{AlN} + (1-x)P_{sp}^{GaN}$

III-N Piezoelectric polarization considerations

GaN lattice parameter [2]: $a_{GaN} = 0.316\ nm$

AlN lattice parameter [2]: $a_{AlN} = 0.311\ nm$

Y. Baines, J. Buckley, J. Biscarrat, G. Garnier, M. Charles,
W. Vandendaele, C. Gillot and M. Plissonnier.

Al$_x$Ga$_{1-x}$N lattice parameter: $a_{Al_xGa_{1-x}N} = xa_{AlN} + (1-x)a_{GaN}$

GaN elastic constants [2]: $c_{13}^{GaN} = 106\ GPa$ *and* $c_{33}^{GaN} = 398\ GPa$

AlN elastic constants [2]: $c_{13}^{AlN} = 99\ GPa$ *and* $c_{33}^{AlN} = 389\ GPa$

Al$_x$Ga$_{1-x}$N elastic constants: $c_{13}^{Al_xGa_{1-x}N} = xc_{13}^{AlN} + (1-x)c_{13}^{GaN}$ *and*

$$c_{33}^{Al_xGa_{1-x}N} = xc_{33}^{AlN} + (1-x)c_{33}^{GaN}$$

GaN piezoelectric coefficients [2]: $e_{31}^{GaN} = -0.33\ C/m^2$ *and* $e_{33}^{GaN} = 0.65\ C/m^2$

AlN piezoelectric coefficients [2]: $e_{31}^{AlN} = -0.58\ C/m^2$ *and* $e_{33}^{AlN} = 1.58\ C/m^2$

Al$_x$Ga$_{1-x}$N piezoelectric coefficients: $e_{31}^{Al_xGa_{1-x}N} = xe_{31}^{AlN} + (1-x)e_{31}^{GaN}$ *and*

$$e_{33}^{Al_xGa_{1-x}N} = xe_{33}^{AlN} + (1-x)e_{33}^{GaN}$$

Al$_x$Ga$_{1-x}$N piezoelectric charge [1]:

$$P_{pz}^{Al_xGa_{1-x}N} = 2\frac{a_{GaN}-a_{Al_xGa_{1-x}N}}{a_{Al_xGa_{1-x}N}}\left(e_{31}^{Al_xGa_{1-x}N} - e_{33}^{Al_xGa_{1-x}N}\frac{c_{13}^{Al_xGa_{1-x}N}}{c_{33}^{Al_xGa_{1-x}N}}\right)$$

AlGaN/AlN/GaN heterojunction surface charges (see manuscript notations):

- *AlN/GaN interface:* $\sigma_{10} = P_{sp}^{AlN} + P_{pz}^{AlN} - P_{sp}^{GaN}$
- *AlGaN/AlN interface:* $\sigma_{21} = P_{sp}^{Al_xGa_{1-x}N} + P_{pz}^{Al_xGa_{1-x}N} - P_{sp}^{AlN} - P_{pz}^{AlN}$

III-N carrier masses:

Free electron mass: m_0

GaN electron effective mass: $m_e^{GaN} = 0.22\ m_0$

GaN hole effective mass: $m_h^{GaN} = 1\ m_0$

AlN electron effective mass: $m_e^{AlN} = 0.35\ m_0$

Al$_x$Ga$_{1-x}$N electron effective mass: $m_e^{Al_xGa_{1-x}N} = xm_e^{AlN} + (1-x)m_e^{GaN}$

Y. Baines, J. Buckley, J. Biscarrat, G. Garnier, M. Charles,
W. Vandendaele, C. Gillot and M. Plissonnier.

WKB approximation compared to Transfer Matrix formalism

In the following we recall the recessed diode's forward current recorded at 300K and superimpose the calculated currents by computing the transmission probability of the system using:

- *The WKB approximation on the one hand*
- *The Transfer Matrix formalism on the other hand*

Note that all parameters are kept constant, only the calculation methods to account for the electron transmission probability as a function of energy is varied.

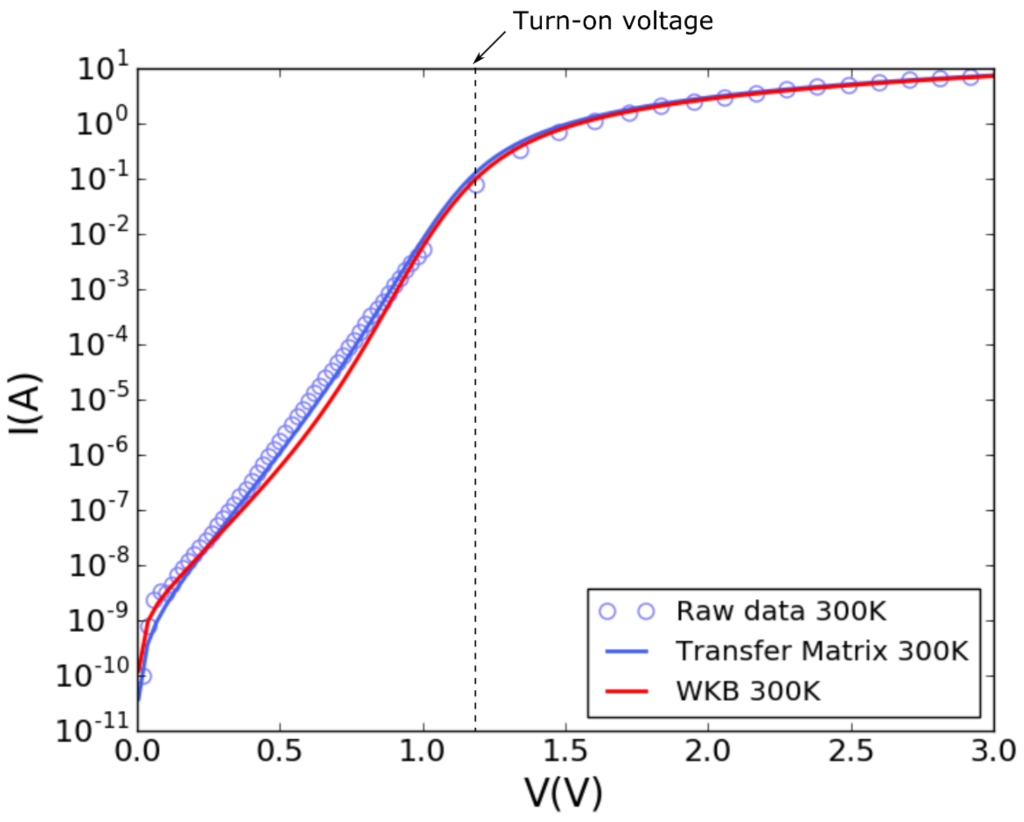

Figure 1: Forward conduction I(V) characteristics of the recessed diode recorded at 300K and comparison with calculated currents using the WKB approximation and the Transfer Matrix formalism.

Y. Baines, J. Buckley, J. Biscarrat, G. Garnier, M. Charles, W. Vandendaele, C. Gillot and M. Plissonnier.

Figure 1 indicates that both calculations are close, however the WKB approximation is less efficient in the reproduction of the tunnel current under the turn-on voltage. As can be observed, the current is underestimated compared to the Transfer Matrix calculation. The authors believe that this feature relates to the lower accuracy of the WKB approximation when estimating the transmission probability of an arbitrary shaped potential barrier. In comparison the Transfer Matrix approach is more rigorous, which is a known feature, although it is more time demanding and requires specific attention to avoid numerical instabilities.

References:

[1] Ambacher, O. *et al.,* Two-Dimensional electron gases induced by spontaneous and piezoelectric polarization charges in N- and Ga-face AlGaN/GaN heterostructures. *J. Appl. Phys.* **85**, **6**, 3222-3233, (1999).

[2] http://www.ioffe.ru/SVA/NSM/Semicond/.
